# Calli Essential Oils Synergize with Lawsone against Multidrug Resistant Pathogens

**DOI:** 10.3390/molecules22122223

**Published:** 2017-12-20

**Authors:** Sameh S. M. Soliman, Abrar I. Alsaadi, Eman G. Youssef, Gregory Khitrov, Ayman M. Noreddin, Mohamed I. Husseiny, Ashraf S. Ibrahim

**Affiliations:** 1 Sharjah Institute for Medical Research, and College of Pharmacy, University of Sharjah, P.O. Box 27272, Sharjah, UAE; anoreddin@sharjah.ac.ae; 2 Faculty of Pharmacy, Zagazig University, Zagazig 44519, Egypt; melsayed@coh.org; 3 Division of Infectious Diseases, Los Angeles Biomedical Research Institute, Harbor-UCLA Medical Center, Torrance, CA 90509, USA; aalsaadi1@toromail.csudh.edu (A.I.A.); emangouda@psas.bsu.edu.eg (E.G.Y.); 4 Department of Biotechnology & Life Sciences, Faculty of Postgraduate Studies for Advanced Sciences (PSAS), Beni-Suef University, Beni-Suef, Egypt; 5 Molecular Instrumentation Center, Department of Chemistry, University of California Los Angeles, Los Angeles, CA 90095, USA; khitrov@chem.ucla.edu; 6 East Virginia Medical School Family Practice, Norfolk, VA 23507, USA; 7 Department of Translational Research & Cellular Therapeutics, Beckman Research Institute of City of Hope, Duarte, CA 91010-3000, USA; 8 David Geffen School of Medicine at UCLA, Los Angeles, CA 90095, USA

**Keywords:** antimicrobials, *Calligonum*, essential oil, lawsone, combination treatment, liposomes

## Abstract

The fast development of multi-drug resistant (MDR) organisms increasingly threatens global health and well-being. Plant natural products have been known for centuries as alternative medicines that can possess pharmacological characteristics, including antimicrobial activities. The antimicrobial activities of essential oil (Calli oil) extracted from the *Calligonum comosum* plant by hydro-steam distillation was tested either alone or when combined with lawsone, a henna plant naphthoquinone, against MDR microbes. Lawsone showed significant antimicrobial activities against MDR pathogens in the range of 200–300 µg/mL. Furthermore, Calli oil showed significant antimicrobial activities against MDR bacteria in the range of 180–200 µg/mL, *Candida* at 220–240 µg/mL and spore-forming *Rhizopus* fungus at 250 µg/mL. Calli oil’s inhibition effect on *Rhizopus*, the major cause of the lethal infection mucormycosis, stands for 72 h, followed by an extended irreversible white sporulation effect. The combination of Calli oil with lawsone enhanced the antimicrobial activities of each individual alone by at least three-fold, while incorporation of both natural products in a liposome reduced their toxicity by four- to eight-fold, while maintaining the augmented efficacy of the combination treatment. We map the antimicrobial activity of Calli oil to its major component, a benzaldehyde derivative. The findings from this study demonstrate that formulations containing essential oils have the potential in the future to overcome antimicrobial resistance.

## 1. Introduction

The emergence of MDR microbial strains with the lack of development of new antimicrobials endangers the future management of infectious diseases. Thus, alternatives to traditionally-used antibiotics can be of great benefit in combating MDR pathogens. Compounds isolated from natural sources are alternatives to many drugs, including those with antimicrobial activity, mainly because of their reduced side effects compared to synthetic drugs [1]. Natural compounds can be considered as privileged structures functionally evolved for the purpose of interaction with specific targets. Many attempts have been made to investigate the potential role of plant extracts and their major active compounds to overcome antibiotic resistance. For example, naphthoquinones, including lawsone (2-hydroxy-1,4-naphthoquinone), are promising antibacterial and antifungal compounds [2,3,4]. Lawsone is the principal active ingredient of the *Henna* plant [5,6]. Although lawsone is known as an antimicrobial compound, it’s effect is not consistent when tested against different microbes including *Candida* and spore-forming fungi [7]. Furthermore, lawsone is not stable and shows a degradation behavior over time [8]. 

Essential oils are also products of the secondary metabolism of aromatic plants. Essential oils are known to possess different biological properties including antimicrobial activities [9]. Essential oils are multi-component products and, hence, can exert greater antimicrobial activity compared to their major components alone [9]. Due to the multi-component nature of essential oils, antimicrobial drug resistance is less likely to be developed since these oils affect numerous targets in the pathogen [10]. Consistent with this hypothesis, clinical resistance to essential oil has not yet been reported [11]. Essential oils exert their antimicrobial effect mainly by affecting membrane permeability [12] due to their lipophilic nature [13]. 

*Calligonum comosum* is an aromatic plant reported as a source of essential oil [14]. Although the plant extract shows antimicrobial activities [15,16,17], its antimicrobial properties have never been attributed to its oil content. Furthermore, the broad spectrum antimicrobial activities and the active components of the oil extracted from United Arab Emirates (UAE) *Calligonum* plant have never been investigated.

In this study, we investigated the antimicrobial activities of lawsone from *Henna* and Calli oil from *Calligonum comosum*, alone or in combination, against several pathogens known for their resistance to antibiotics. We also investigated the toxicity of these plant extracts with or without liposome preparations. We report on the broad-spectrum activity of both lawsone and Calli oil against methicillin-resistant *Staphylococcus aureus* (MRSA), Gram-negative MDR bacteria, *Candida* species (including *C. auris*), and the spore-forming *Rhizopus* fungus, the major cause of the lethal infection mucormycosis. We also show the enhanced activity of combination of lawsone and Calli oil against these priority pathogens. Finally, liposome preparations of lawsone and Calli oil are less toxic to mammalian cells. 

## 2. Results

### 2.1. The Anti-Microbial Effect of Lawsone and Calli Oil

#### 2.1.1. Lawsone is a Potential Antimicrobial Candidate

**Figure 1:**
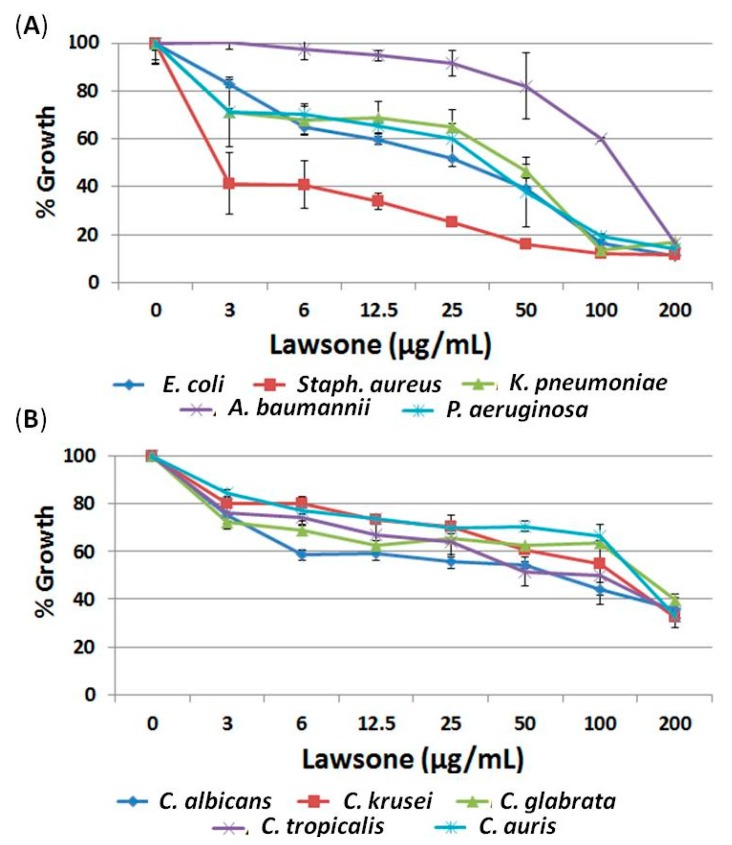
Antibacterial and anti-*Candida* effects of lawsone. The effect of different lawsone concentrations on (**A**) Gram-negative and Gram-positive bacteria, and (**B**) different *Candida* spp. Lawsone at 200 µg/mL caused significant inhibition of the growth of all tested bacterial spp. and *Candida* strains down to ~>90% and 60%, respectively. The data display the mean of the percentage of microbial growth at different concentrations ± standard error of the mean.

The potential antimicrobial activity of lawsone was evaluated against several priority MDR pathogens. Lawsone showed strong cidal activity against Gram-positive MRSA and Gram-negative bacteria including MDR *Pseudomonas aeruginosa*, *Klebsiella pneumoniae* carbapenemase (KPC)-producing bacteria, extensively drug-resistant *Acinetobacter baumannii*, and *E. coli* (Figure 1A). Similarly, lawsone demonstrated cidal activity against *Candida* species including *C. albicans* (SC5314), *C. krusei*, *C. glabrata*, *C. tropicalis*, and the MDR *C. auris* (CAU09) (Figure 1B). The growth inhibition was concentration-dependent with a range of 100–200 µg/mL being sufficient to significantly inhibit the growth of all tested bacteria spp. Lawsone at 200 µg/mL caused ~90%, 88%, 92%, 94% and 96% inhibition of *E. coli*, MRSA, KPC-producing bacteria, *A. baumannii*, and *P. aeruginosa*, respectively (Figure 1A) compared to a 100% inhibition due to 0.7–10 µg/mL colistin for Gram-negative bacteria and 3 µg/mL vancomycin for MRSA (Supplementary Table S1). On the other hand, 200 µg/mL lawsone resulted in ~60% inhibition of the growth of all tested *Candida* spp. (Figure 1B) compared to a 100% inhibition due to 1–3 µg/mL ketoconazole (Supplementary Table S2). The effective MIC of lawsone for bacteria strains and *Candida* was 220–240 and 250–300 µg/mL, respectively (Supplementary Tables S1 and S2).

#### 2.1.2. *Calligonum* Plant Essential Oil (Calli Oil) Showed Potential Antimicrobial Activities

**Figure 2:**
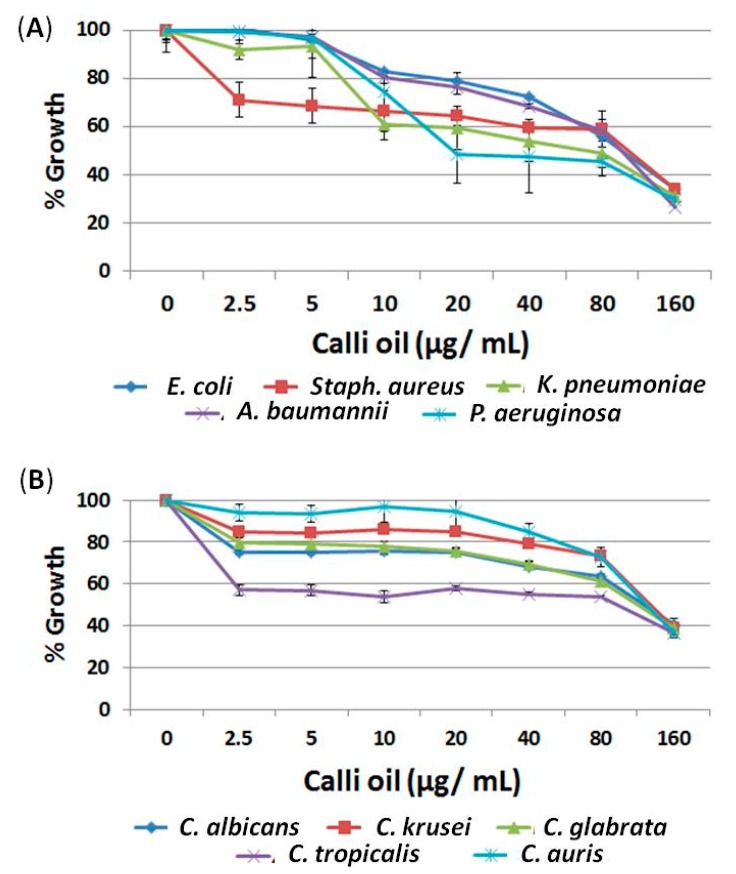
Antibacterial and anti-*Candida* effects of Calli oil isolated from *Calligonum comosum* grows in the UAE. The effect of different Calli oil concentrations on (**A**) Gram-negative and Gram-positive bacteria and (**B**) different *Candida* spp. Calli oil at 160 µg/mL caused significant inhibition of the growth of all tested bacterial spp. and *Candida* strains down to ca. >60%. The data display the mean of the percentage of microbial growth at different concentrations ± standard error of the mean.

Calli oil isolated from *Calligonum comusum* (Arta) plant by hydro-steam distillation showed broad spectrum antimicrobial activities. At a concentration of 160 µg/mL, the extracted oil demonstrated 66–71% inhibition of all tested bacterial spp. compared to untreated controls (Figure 2A). Similarly, Calli oil inhibited the growth of all tested *Candida* spp. including *C. albicans* (SC5314), *C. krusei*, *C. glabrata*, *C. tropicalis*, and *C. auris* (CAU09) (Figure 2B). The inhibition was also concentration-dependent with 160 µg/mL significantly reducing the yeast growth by ~60%. The MIC of Calli oil was determined as 180–200 µg/mL for the bacterial strains and 220–240 µg/mL for *Candida* spp. (Supplementary Tables S1 and S2).

**Figure 3:**
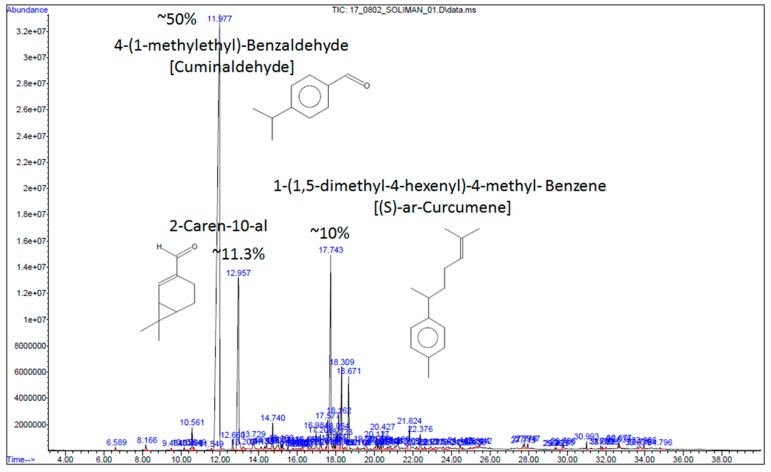
GC-MS analysis of Calli oil. The analysis showed that 4-(1-methylethyl)-benzaldehyde, 2-caren-10-al and 1-(1,5-dimethyl-4-hexenyl-4-methyl-benzene were the three major components of Calli oil. Y axis represents the abundance of the peaks.

The amount of Calli oil extracted from *Calligonum* plant was calculated as 0.4 µg oil/gm plant (dry weight) and the major components were 4-(1-methylethyl)-benzaldehyde (cuminaldehyde, 50%), 2-caren-10-al (11.3%), and 1-(1,5-dimethyl-4-hexenyl-4-methyl-benzene (curcumene, 10%) identified by GC-MS analysis (Figure 3).

### 2.2. The Synergistic Effect of both Lawsone and Calli Oil

#### 2.2.1. Calli Oil Augmented the Antimicrobial Activities of Lawsone

**Figure 4:**
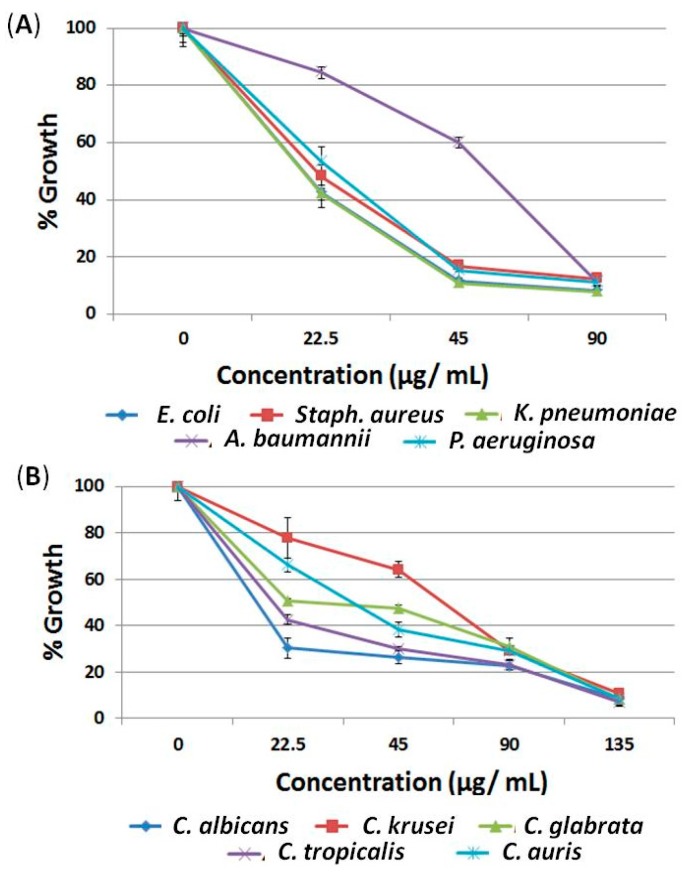
Antibacterial and anti-*Candida* effects of combined treatment using Calli oil and lawsone. The effects of different concentrations of combined substances on (**A**) Gram-negative and Gram-positive bacteria and (**B**) different *Candida* spp. Combination of lawsone and Calli oil showed a synergistic effect on MDR microbes with 90 µg/mL and 135 µg/mL caused more than 90% inhibition of tested bacterial spp. and *Candida* spp., respectively. The data display the mean of the percentage of microbial growth at different concentrations ± standard error of the mean.

Preliminary study by mixing lawsone and Calli oil showed antimicrobial activities that exceeded the activity of each product alone. Briefly, a mixture of 50 µg/mL lawsone and 40 µg/mL Calli oil (for a total of 90 µg/mL) caused ≥90% growth inhibition of all tested bacterial strains (Figure 4A). Similarly, a mixture of 75 µg/mL lawsone and 60 µg/mL Calli oil (for a total of 135 µg/mL) caused >90% growth inhibition of all tested *Candida* spp. (Figure 4B). The MIC of the combination preparation against the tested bacteria, or *Candida* ranged between 100–150 µg/mL, a ~50% reduction in the MIC values of each product alone (Supplementary Tables S1 and S2).

#### 2.2.2. Calli Oil Enhanced the Antifungal Activities of Lawsone against *Rhizopus* Fungus

**Figure 5:**
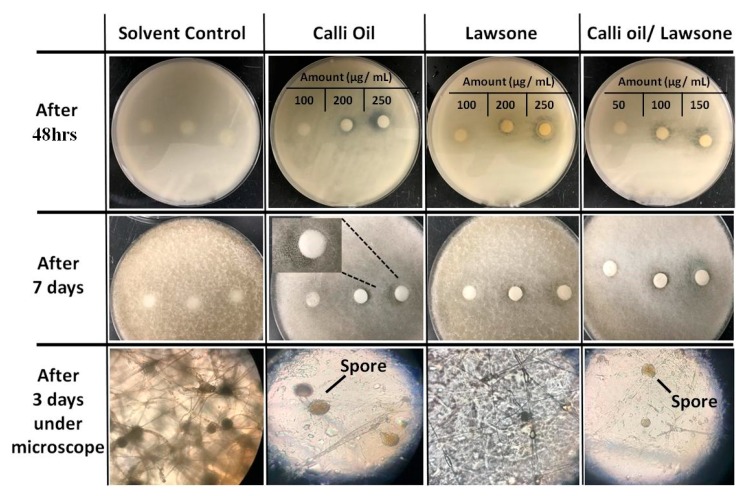
The antifungal effect of lawsone, Calli oil and combined Calli oil/lawsone on *R. delemar*, a spore-forming fungus at different concentrations. The effect was measured visually by disc diffusion assay and under light microscope. The experiment was repeated at least three times.

Due to the enhanced efficacy of combination of Calli oil and lawsone against MDR bacteria and *Candida*, the combined preparation was tested against spore-forming fungus, *Rhizopus delemar*. *R. delemar* PDA cultures were treated with either lawsone, or Calli oil, at concentrations 100, 200, and 250 µg/mL. Similarly, *R. delemar* cultures were treated with combined Calli oil/lawsone at concentrations 50, 100 and 150 µg/mL. All cultures were incubated for 48 h in the dark at 37 °C followed by measuring inhibition zone of growth using disc diffusion assay. Lawsone, Calli oil, and their combined preparation caused significant inhibition zone of growth corresponding to ~18 ± 0.75, 23 ± 0.5, and 20 ± 0.43 mm in diameter, respectively (Figure 5). The antimicrobial effects of tested substances on *Rhizopus* fungus was extended for more than seven days, where the oil alone caused the appearance of white spores at the zone of inhibition, lawsone showed less sporulated thinner hyphae and the combined treatment caused less number of white spores, but thinner hyphae (Figure 5).

### 2.3. The Effect of Liposome Preparation on the Antimicrobial Activity of Lawsone and Calli Oil

Liposome preparation enhanced activities, reduced the toxicity of both natural products, and promoted the stability of lawsone.

#### 2.3.1. Liposome Preparation Enhanced the Antimicrobial Activity of Natural Product Employed

Liposomes made of lawsone and Calli oil by the ether injection method caused significant growth inhibition to bacteria and *Candida* similar to their combination treatment (Supplementary Tables S1 and S2). Measuring the amount of liposome-incorporated Calli oil (by GC-MS) and lawsone (by LC-MS) indicated that ~87% of the combined oil was included (calculated by measuring the cuminaldehyde content of the oil) and only ~76% of lawsone was incorporated in the final liposomes preparation. These results indicate that Calli oil combined with lawsone with or without liposome preparation enhanced the antimicrobial activities over each natural product alone by ca. three-fold versus bacteria and ca. four-fold versus *Candida*. On the other hand, liposome preparation showed similar antimicrobial pattern measured by disc diffusion assay up to one month of storage at room temperature; compared to reduction in antimicrobial activity of lawsone alone with time (data not shown). The results indicated that liposomes preparation promoted the stability of lawsone. 

#### 2.3.2. Liposome Preparation Enhanced the Anti-*Rhizopus* Activity of Natural Product Employed

**Figure 6:**
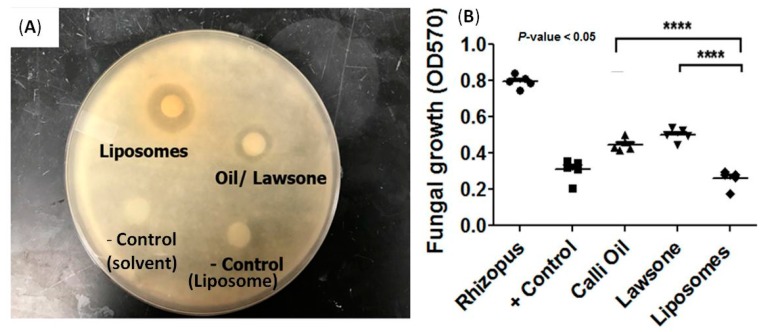
The antifungal effect of liposomes compared to the combined treatment and individual Calli oil or lawsone on *R. demelar* fungus. Comparison between liposomes prepared by mixing equal amounts of Calli oil and lawsone and combined amount of both substances on *Rhizopus* fungus using (**A**) disc diffusion assay and (**B**) microdilution assay. The data display the mean of the growth of *Rhizopus* ± standard error of the mean. The statistical significance was calculated with one-way ANOVA and significance level indicated by asterisks (**** *p* = 0.0001).

Liposomes preparation of Calli oil/lawsone showed significant growth inhibition (zone of inhibition, 30 mm diameter) compared to combination treatment (20 mm diameter) (Figure 6A). Liposome preparation caused a significant (*p* = 0.0001) reduction in the fungal growth greater than, or equal to, two-fold compared to each substance alone measured by microdilution assay (Figure 6B). The inhibitory concentrations of lawsone, Calli oil, combined treatment and liposome against *R. delemar* were 250 µg/mL, 250 µg/mL, 150 µg/mL (i.e., 80 µg/mL lawsone and 70 µg/mL Calli oil) and ~150 µg/mL compared to 5 µg/mL amphotericin B as the positive control. 

#### 2.3.3. Liposome Preparation Significantly Reduced the Toxicity of the Natural Product Employed

**Figure 7:**
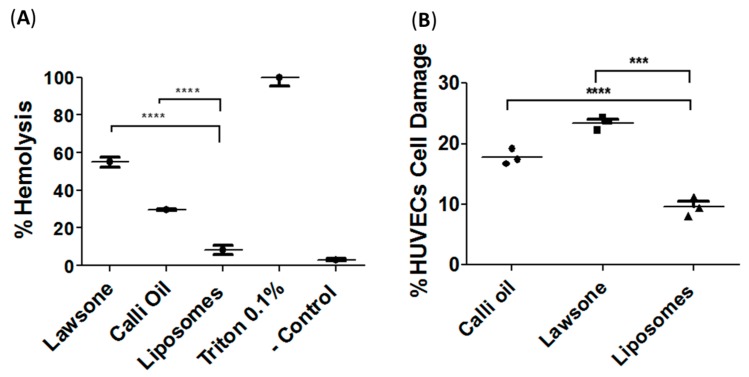
Cytotoxicity assays of prepared liposomes compared to Calli oil or lawsone alone using either (**A**) RBCs hemolysis assay or (**B**) HUVEC injury assay. The data display the mean of the percentage of hemolysis or percentage of cell damage ± standard error of the mean. The statistical significance was calculated with one-way ANOVA and significance level indicated by asterisks (*** *p* = 0.0005, and **** *p* = 0.0001).

The cytotoxic effect of lawsone, Calli oil, or their combination, was compared using both hemolysis of red blood cells (RBCs) and mammalian cell injury assays. Liposome preparation of lawsone and Calli oil was significantly (*p* = 0.0001) less toxic when compared to each natural product alone (ca. seven- and four-fold reduction in hemolytic toxicity versus lawsone or Calli oil, respectively) (Figure 7A). Furthermore, liposome caused significant (*p* = 0.0001 and *p* = 0005) reduction in damage to human umbilical vein endothelial cells (HUVECs) (~2.5-fold reduction in damage when compared to each product alone) (Figure 7B).

## 3. Discussion

Lawsone showed potential antimicrobial activities against Gram-positive and Gram-negative bacteria, *Candida* and spore-forming fungi within the range of 200–300 µg/mL. The antimicrobial activity of lawsone is partially lower than the results obtained by Rahmoun, et al., 2012 [4]. The minimum amount required for inhibition is different than what has been reported possibly because of the degradation behaviors of lawsone [8].

Consistent with previous reports [14], we found that *Calligonum comosum* collected from the UAE desert is a good source of essential oils. Although the plant extract is known to have antimicrobial activity [15], to our knowledge this study is the first to attribute this antimicrobial activity to *Calligonum* oil (Calli oil) against MDR pathogens including Gram-positive and Gram-negative bacteria, *Candida* species, and the Mucorales mold, *R. delemar*. Another different finding from our studies than previously reported studies lies in the composition of oils extracted from UAE *Calligonum comosum*. The major components of the oil isolated in our study were 4-(1-methylethyl)-benzaldehyde (cuminaldehyde), 2-caren-10-al and 1-(1,5-dimethyl-4-hexenyl-4-methyl-benzene (curcumene), whereas in a previous study essential oil from the same plant was reported as lauric, myristic, and palmitic acids [14]. This difference could be due to the geographical differences from where the plants were obtained [18]. It is prudent to mention that the potential antimicrobial activities of benzaldehyde and cuminaldehyde were previously realized [19,20]. 

One critical finding of our studies is the activity seen with Calli oil against the Mucorales mold, *R. delemar*, a rare, but lethal, fungal infection. It appears in addition to the direct effect of the Calli oil on the growth of the spore-forming mold, it appears the oil caused discoloration of the black spores potentially due to prevention of melanin formation [21]. Melanin’s role in virulence of fungal pathogens, including the ability to resist phagocyte killing is well-described [21]. Thus, Calli oil has the potential to directly kill this lethal mold and also enhance the immune system to clear the infection.

Synergistic combination between conventional antibiotics and essential oils is currently under investigation and can represent a potential area for future novel treatment regimens in combating antimicrobial resistance [22]. Such combination treatment has the potential to surpass monotherapy by producing enhanced antimicrobial activity [23]. Our studies of combining lawsone and Calli oil clearly support this concept and represent a future promising novel treatment for MDR pathogens. 

Liposomes are spherical vesicles consisting of outer lipid bilayers surrounding aqueous core [24]. Hydrophilic antibiotics can be encapsulated in the internal aqueous compartment, whereas hydrophobic drugs may bind to or incorporate in the lipid bilayer [25]. Liposome encapsulation of antibiotics can increase the therapeutic index of antibiotics by augmenting their concentrations at the site of infection and reducing their toxicity [26]. Similarly, we found that the activity of liposomes prepared from lawsone and Calli oil was enhanced and at lower doses. We also found that these liposome preparations were less toxic than the natural products and provided stability over a long period of time. We hypothesize that the liposome preparation of using essential oil within the lipid layer likely potentiates the antimicrobial activities since both the hydrophobic outer layer and the inner aqueous core have antimicrobial activities. A multi-component outer oily layer will disturb the microbial membrane and, hence, enhances and stabilizes the effect of aqueous core-containing lawsone. This approach is also likely to improve treatment of severe infections since liposomes can achieve a significant longer blood and tissue half-life [27]. Consistent with Sherry, et al. [28], the entrapped oil was greater than the entrapped aqueous substance mainly because the oil entrapped with the lipid layer and, hence, stabilized its presence; however, lawsone in the aqueous core may be leaked from the prepared liposomes.

As concluding remarks, the formulation of antimicrobial liposomes by combining both hydrophilic natural products and hydrophobic essential oil can be a future promising area for improved delivery of naturally present products with antimicrobials activity against MDR-resistant pathogens. The multi-functional entrapped essential oil in these liposomes can stabilize the structure, increase the interaction of the liposome with the hydrophobic surface of pathogens, and directly affect the viability of the targeted microbes. Additionally, and as noted before [29] these liposomes can reduce any potential toxicity of the delivered drugs and increase their half-life in plasma and targeted tissues.

## 4. Materials and Methods

### 4.1. Materials

Lawsone, colistin, vancomycin, ketoconazole, and amphotericin B were all purchased from Sigma (Sharjah, UAE).

### 4.2. Essential Oil Extraction

The air-dried *Calligonum comosum* aerial parts were collected from the desert of Sharjah, UAE. The plant was ground to obtain a homogeneous powder and subjected to hydro-steam distillation [30] for 3 h. Collected oil was dried over anhydrous sodium sulphate and then stored at 4 °C in sealed vials before antimicrobial testing and GC-MS analysis.

### 4.3. Studying the Antimicrobial Activities of Tested Substances

The antibacterial activity of lawsone and Calli oil was studied against methicillin-resistant *Staphylococcus aureus* (MRSA) strain and Gram-negative bacteria including *P. aeruginosa*, *E. coli*, and the multidrug resistant (MDR) *A. baumannii* and *Klebsiella pneumoniae* on agar plates and in liquid broth media according to a modified version of Clinical and Laboratory Standards Institute (CLSI) [31]. Briefly, 0.1 mL containing 10^5^ CFU/mL was spread on Luria-Bertani (LB) agar plates [32]. The plates were then incubated at 37 °C with filter discs (8 mm diameter) saturated with different dilutions of lawsone (3, 6, 12, 25, 50, 100 and 200 µg/mL), Calli oil (2.5, 5, 10, 20, 40, 80 and 160 µg/mL) and their combination (12.5, 25, 50 and 75 μg/mL) for 24 h. For the microdilution method, the microbial strains were incubated with the aforementioned concentrations of substances into LB broth media inoculated with 10^5^ CFU/mL in 96-well microplates at 37 °C for 24 h and the microbial growth (turbidity) was measured by microplate reader (DYNEX Technologies, Chantilly, VA, USA) at OD_600_. Each test was performed in triplicate. The anti-*Candida* activities were similarly measured against *C. albicans* (SC5314), *C. krusei*, *C. glabrata*, *C. tropicalis*, and *C. auris* (CAU09) and according to a modified version of Clinical and Laboratory Standards Institute (CLSI) (Wayne, PA, USA) [31] using LB agar plates for disc diffusion assay or yeast nitrogen base (YNB) supplemented with 100 mM glucose for microdilution assay.

For the spore-forming fungus (*Rhizopus delemar*), the antimicrobial activities were determined by using the reference procedure of the Antifungal Susceptibility Testing Subcommittee of EUCAST for spore-forming molds [33]. Briefly, flat-bottom microdilution 96 well-plates were loaded with 200 µL RPMI 1640 medium supplemented with 2% glucose and an inoculum of 2 × 10^5^ CFU/mL. Growth inhibition was visually determined at 24, 48 and 72 h. The reading was performed after 5 min of agitation on a microdilution plate shaker with a spectrophotometer (DYNEX Technologies, Chantilly, VA, USA) at 570 nm.

Colistin, vancomycin, ketoconazole, and amphotericin B were used as positive controls against Gram negative bacteria, Gram-positive bacteria, *Candida* and fungi, respectively. Cultures without antimicrobials served as negative controls. All experiments were repeated in triplicate. All microbial strains are clinical isolates from patients who were seen at Harbor-UCLA Medical Center, Torrance, CA, USA. The antimicrobial activities of all substances were tested either by disc diffusion assay, microdilution assay or by measuring the minimum inhibitory concentration (MIC).

### 4.4. Stability Testing

Calli oil, lawsone, and their liposome preparation were stored separately at room temperature (~25 °C) for one month. The antimicrobial activity of each substance alone and in liposome preparation was measured by disc diffusion assay at different time intervals. The results obtained were compared to those obtained prior to storage. The diameter of zone of growth inhibition (in mm) was read at 24 h.

### 4.5. Cytotoxicity Assay

The cytotoxic assays of tested substances were performed by hemolysis assay and mammalian cell damage assay as reported below.

#### 4.5.1. Hemolysis Assay

Each tested substance was measured as the amount of hemoglobin released by the lysis of human erythrocytes [34,35]. Briefly, fresh whole blood from healthy individual was collected into heparinized vacutainer from Harbor-UCLA Hospital and 1 mL whole blood was immediately centrifuged at 500× *g* for 10 min using a benchtop centrifuge (Eppendorf 5804R refrigerated benchtop, Pittsburgh, PA, USA). The erythrocytes were washed three times with DPBS supplemented with 1 mg/mL bovine serum albumin (BSA) and then re-suspended to 3 × 10^7^ cells/mL in DPBS. Washed cells (3 × 10^6^ cells per well) were incubated with the substance dissolved in the washing buffer at different concentrations (ranging from 100 to 200 μg/mL) in round-bottomed 96-well plates in a final volume of 200 μL. Washing buffer and 0.1–1% Triton X-100 were used as negative and positive controls, respectively. The plate was incubated at 37 °C for 30 min, followed by 30 min incubation on ice, and the intact cells were precipitated by centrifugation at 500× *g* for 10 min at 4 °C and the supernatants (125 μL) were transferred to a flat-bottom 96-well plate to measure hemoglobin release by absorbance at 405 nm using a microplate reader. The absorbance values for each sample were subtracted from the absorbance value obtained for washing buffer-treated cells and the hemolytic activity (%) was calculated. The experiment was conducted in triplicate and the data was analyzed using two-way analysis of variance (ANOVA). The 50% cytotoxic concentration (CC_50_) values were calculated as the concentration of substance caused 50% hemolysis compared to 100% hemolysis of erythrocytes treated with 0.1% triton X-100. Written informed consent was obtained from donor for the use of his/her blood. All experimental procedures were approved by Institutional Review Board (IRB) of LA Biomed under protocol 11671-11.

#### 4.5.2. Mammalian Cell Damage Assay

Human umbilical vein endothelial cells (HUVEC) damage were quantified using a ^51^Cr release assay [36]. Briefly, cells grown in 96-well tissue culture plates containing detachable wells were incubated with 1 μCi/well Na_2_^51^CrO_4_ (ICN) in M-199 medium for 16 h. On the day of the experiment, the unincorporated ^51^Cr was aspirated, and wells were washed twice with pre-warmed HBSS. Cells were treated with substances suspended in RPMI 1640 medium supplemented with glutamine and incubated at 37 °C in a 5% CO_2_ incubator. Spontaneous ^51^Cr release was determined by incubating the cells only in culture medium supplemented with glutamine. After 16 h incubation, the medium was aspirated from each well and transferred to glass tubes, and cells were manually detached and placed into another set of tubes. The amount of ^51^Cr in the aspirate and the detached well was determined by gamma counting. The total amount of ^51^Cr incorporated by the cells in each well was calculated as the sum of radioactive counts per min of the aspirated medium and radioactive counts of the corresponding detached wells. After data were corrected for variations in the amount of tracer incorporated in each well, the percentage of specific cell release of ^51^Cr was calculated as follows: ((experimental release) − (spontaneous release))/(1 − (spontaneous release)). Each experimental condition was tested in triplicate, and the experiment was repeated twice.

### 4.6. Gas Chromatography-Mass Spectrometry (GC-MS)

GC-MS measurements were carried out using an Agilent model 7683 (Wilmington, DE, USA) autosampler, 6890 gas chromatograph, and 5975 inert mass selective detector in the electron impact (EI) mode. EI energy was set to 70 eV. Separation was carried out on an Agilent HP5-MS column with dimensions 30 m × 250 µm × 0.25 µm. Ultra-high purity grade He (Airgas, Kennesaw, GA, USA) was used as carrier gas with the flow set to 0.8 mL/min in constant flow mode. The initial oven temperature was set to 45 °C for 1 min followed by a 30 °C/min ramp to a final temperature of 300 °C which was maintained for 3 min. A 3.2 min solvent delay was used. The MSD was set to scan the 40–1050 *m*/*z* range. Data collection and analysis were performed using MSD Enhanced Chemstation software (G1701EA, Agilent, Wilmington, DE, USA). Product spectra were identified by comparison of the measured fragmentation patterns to those found in the NIST 08 Mass Spectral Library.

### 4.7. Liquid Chromatography-Mass Spectrometry (LC-MS)

For LC-MS quantification of lawsone, prepared liposomes were dissolved in methanol and 10 µL was injected in to LC-MS. LC-MS analyses were carried out in negative ion mode by electrospray ionization (ESI) on a ACQUITY UPLC triple quadrupole (Xevo TQD, Waters, Milford, MA, USA) instrument equipped with MassLynx software (4.1, Waters, Milford, MA, USA ). The solvent system was (A: 100% acetonitrile and B: water containing 0.1% formic acid). The solvent gradient was 0–10 min/75% A, 8 min/100% A, 2 min/75% A and 3 min/75% A. The flow rate was 0.3 mL/min and the injection volume was 10 µL. All solvents and reagents were HPLC grade and used without further purification.

### 4.8. Liposome Preparation and Drug Loading Using Solvent Dispersion Ether Injection (Solvent Vaporization) Method

Phospholipids (100 mg/mL) and essential oil (80 µg/mL) isolated from *Calligonum* were dissolved in diethyl ether and then vortexed for 5 min. The solution was then gradually injected to an aqueous solution of lawsone (100 µg/mL) at 55 °C. The ether was then removed under vacuum [37,38] and the solution left was centrifuged at 10,000× *g* for 15 min, followed by aspiration of water and vacuum dried.

### 4.9. Statistical Analysis

The data was collected and graphed using Microsoft Excel and Graph Pad (5.04, La Jolla, CA, USA) for Windows for statistical analysis. The effects of individual natural substance or in combination on MDR bacteria and *Candida* spp. inoculated onto solid agar media and liquid broth was analyzed by one-way analysis of variance (ANOVA) using Dunnett’s Multiple Comparison Test. A *p*-value < 0.05 was considered as significant.

## Supplementary Materials

Tables indicating the MIC of all tested substances are available online.
