# Supplementary material for: Calli Essential Oils Synergize with Lawsone against Multidrug Resistant Pathogens
Source: Molecules. 2017 Dec 20;22(12):2223. doi: 10.3390/molecules22122223 (PMC6034512; doi:10.3390/molecules22122223)
Supplement: Supplementary file 1 [file molecules-22-02223-s001.pdf]

Supplementary Table S1. Minimum inhibitory concentration (MIC) values in µg/mL of lawsone, Calli oil, combined treatment and liposomes against different multidrug resistant bacterial strains after 24hrs incubation period. MIC is the lowest concentration of substance that resulted in 90-100% inhibition of microbial growth.

Bacteria	MIC (µg/mL)					
	**Lawsone**	**Calli oil**	**Combination (1.3 lawsone: 1 oil)**	**Liposome (1 lawsone : 1.2 oil)***	**Colistin**	**Vancomicin**
E. coli	*220*	*200*	*100*	*100*	*2.5*	*NA*
MRSA	*220*	*180*	*100*	*100*	*NA*	*3*
K. pneumoniae	*220*	*180*	*100*	*100*	*10*	*NA*
A. baumannii	*240*	*200*	*110*	*100*	*1.25*	*NA*
P. aeruginosa	*220*	*180*	*100*	*100*	*0.7*	*NA*

*The amount of lawsone and oil measured in the liposome preparation was ~0.8 and 0.9 of the amount employed at the beginning of preparation, respectively. NA= not applicable

Supplementary Table S2. Minimum inhibitory concentration (MIC) values in µg/mL of lawsone, Calli oil, combined treatment and liposomes against different MDR *Candida* strains and *Rhizopus* after 24hrs incubation period. MIC is the lowest concentration of substance that resulted in 90-100% inhibition of microbial growth.

Fungi	MIC (µg/mL)					
	**Lawsone**	**Calli oil**	**Combination (1.3 lawsone: 1 oil)**	**Liposome (1 lawsone : 1.2 oil)***	**Ketoconazole**	**Amphotricin B**
C. albicans	*250*	*220*	*150*	*150*	*1*	*NA*
C. krusei	*300*	*240*	*150*	*150*	*3*	*NA*
C. glabrata	*250*	*220*	*150*	*150*	*1*	*NA*
C. tropicalis	*250*	*220*	*150*	*150*	*1*	*NA*
C. auris	*300*	*240*	*150*	*150*	*3*	*NA*
Rhizopus	*250*	*250*	*150*	*150*	*NA*	*5*

* The amount of lawsone and oil measured in the liposome preparation was ~0.8 and 0.9 of the amount employed at the beginning of preparation, respectively. NA= not applicable
